# Factors Influencing the Experience of Breast and Cervical Cancer Screening Among Women in Low- and Middle-Income Countries: A Systematic Review

**DOI:** 10.1200/GO.22.00359

**Published:** 2023-05-04

**Authors:** Edem J. Akoto, Matthew J. Allsop

**Affiliations:** ^1^ Lekma Hospital, Accra, Ghana; ^2^ Academic Unit of Palliative Care, Leeds Institute of Health Sciences, University of Leeds, Leeds, United Kingdom

## Abstract

The persistent high morbidity and mortality from breast and cervical cancer in low- and middle-income countries (LMICs) may be influenced by global disparities in the uptake of screening services. This review sought to synthesize existing evidence to determine factors that influence the experience of women relating to breast and cervical screening in LMICs.

**METHODS:**

A qualitative systematic review of the literature identified through Global Health, Embase, PsycInfo, and MEDLINE. Eligible studies included those outlining primary qualitative research or mixed-method studies with reporting of qualitative findings, detailing women's experiences of involvement with programs for breast or cervical cancer screening. Framework synthesis was used to explore and organize findings from primary qualitative studies and the Critical Appraisal Skills Programme checklist used for quality assessment.

**RESULTS:**

Database searches yielded 7,264 studies for title and abstract screening and 90 full-text articles for screening, with qualitative data from 17 studies and a total of 722 participants included in this review. Four stages influencing experiences of women were generated across both breast and cervical cancer screening approaches, with individual (eg, knowledge of cancer), social (eg, religion, cultural beliefs), and health system (eg, accessibility) factors identified that influence women's initial and subsequent engagement.

**CONCLUSION:**

This study synthesizes existing evidence of factors that influence engagement with breast and cervical cancer screening in LMICs. Evidence-informed recommendations are proposed that may improve the experience of cancer screening in LMICs, with further research necessary to explore their operationalization and impact on cancer care delivery.

## INTRODUCTION

Globally, breast and cervical cancers are highly prevalent, accounting for disability and the premature death of hundreds of thousands of women each year.^1-3^ In low- and middle-income countries (LMICs), breast and cervical cancers receive far less funding, advocacy, and public and political attention when compared with high-income settings.^4^ In LMICs, women with breast and cervical cancers experience poorer access to care, present with more advanced stages of disease, and are disproportionately affected due to vulnerabilities related to gender inequality (eg, societal values and cultural role expectations), poverty, and environmental factors (eg, high prevalence of infections with oncogenic subtypes of the human papillomavirus [HPV] in sub-Saharan Africa [SSA]).^3,5^ With appropriate resources and quality care, around a third of cancers can be prevented and this can be achieved through reduced exposure to modifiable risk factors and early detection of disease.^6^ Approaches, such as cancer screening for women's cancer, have led to global reductions in cancer mortality rates, although this change has not been observed in LMICs.^7^ This may be due to issues including policy that is fragmented and skewed toward the earliest phases of the patient journey (ie, awareness and stakeholder education), with screening, access to treatment and ongoing support, and palliative care remaining limited and underdeveloped.^8^

**CONTEXT**

**Key Objective**
What are the experiences of breast and cervical cancer screening among women in low- and middle-income countries (LMICs)?
**Knowledge Generated**
A range of factors stratified at the levels of individual, social, and health system factors influence the experience of women undergoing breast and cervical cancer screening from research undertaken in LMICs. Key factors that may influence subsequent engagement with screening include communication and compassion of health care professionals, satisfaction with support and counseling, the levels of physical discomfort during procedures, and the duration of time and related anxiety when awaiting test results.
**Relevance**
Evidence-informed recommendations generated from this review can be used to guide refinements in the delivery of screening programmes for women with breast and cervical cancers, fostering positive and continued engagement.

Most LMICs do not have well-developed screening programs or have ineffective screening services and several barriers such as poverty, lack of knowledge, and illiteracy compound problems with the uptake of screening.^7^ Cost-effective screening interventions such as visual inspection (of the cervix) with acetic acid (VIA), HPV testing, and clinical breast examinations by trained health providers have been approved by WHO for use in LMICs in addition to conventional methods such as Papanicolaou (Pap) smear and mammography.^9^ Barriers associated with the uptake of screening services in LMICs have been researched extensively in the literature.^10,11^ An important phenomenon of interest, the experiences during cancer screening have emerged as a barrier in many studies.^9,12^ In most geographical regions including Asia, Latin America, and SSA, previous engagement with screening programs has been mentioned as facilitating or hindering uptake.^12,13^ While a recent review explored quantitative measures of satisfaction with the experiences during breast cancer screening,^14^ no existing reviews have explored the narrative accounts and qualitative experiences of screening and their effect on further engagement. Detailing knowledge generated from primary research on women's experiences of screening services is important to support advocacy for gender parity in worldwide cancer control policy and to determine how delivery of care can be patient-centered.^15^ This review seeks to synthesize evidence on the reported experiences of breast and cervical cancer screening among women in LMICs to determine the following: (1) What are the experiences of breast and cervical cancer screening among women in LMICs? (2) How do experiences of cancer screening programs influence opinions relating to future engagement with the programs? (3) How can an understanding of women's experiences inform the development of breast and cervical cancer screening programs in LMICs?

## METHODS

A qualitative systematic review of the literature was performed to synthesize existing evidence on the reported experiences of breast and cervical cancer screening among women in LMICs. The review was conducted, and reporting aligned with the Preferred Reporting Items for Systematic Reviews and Meta-Analyses (PRISMA) statement.^16^ The study protocol was registered on the PROSPERO register (CRD42022339509).

### Data Sources

The following databases were consulted using the Ovid platform: Global Health, Embase, PsycInfo, and MEDLINE. Database searches were conducted between July 7 and 31, 2022. A comprehensive search strategy was developed and adapted for each database (see the Data Supplement) comprising keyword and medical subject heading aligned to LMIC settings, breast or cervical cancer screening, and qualitative study. We also examined reference lists of the included articles to identify any further eligible articles.

### Study Selection

Eligible studies included those outlining primary qualitative research or mixed-method studies with reporting of qualitative findings, detailing women's (ie, aged ≥18 years) experiences of involvement with programs for breast screening (either clinical breast examinations, ultrasonography, or mammography) or cervical screening (Pap smear, HPV Testing, VIA). Experience was understood as findings that encompass descriptions around the person (ie, experience of health), patient (experience of disease), and user (experience of health services).^17^ Studies were eligible if they recruited participants from LMICs as defined by the 2021 Development Assistance Committee List of the countries and territories eligible to receive official development assistance produced by the Organisation for Economic Co-operation and Development.^18^ Studies were limited to those written in English which was the language spoken by the reviewers.

### Data Collection Process

The results of database searches were exported to EndNote reference manager, and duplicates were removed. The authors independently reviewed the titles and abstracts of all citations in EndNote. Two separate lists of included title and abstracts were exported into Microsoft Excel to determine alignment and differences in included articles. Consensus on inclusion was arrived at through discussion where there were discrepancies between the two reviewers. The inclusion of articles was then determined based on full-text review, assessed independently by the two authors. Uncertainty around the inclusion of articles at the full-text screening stage was resolved through discussion between the two authors, with a third reviewer available should consensus not have been reached.

### Data Extraction

To ensure consistency, data extraction was performed using an adapted data extraction form for qualitative studies^19^ reproduced in Microsoft Excel (see Data Supplement). Information was extracted relating to the study title, year of publication, country, authors, study characteristics, type of screening program, data collection method, sample size, results, and conclusions. Data extraction was undertaken by one author (E.J.A.) with checking completed by a second author (M.J.A.).

### Quality Assessment

The Critical Appraisal Skills Programme Systematic Review Checklist was used for the quality assessment of all included studies (see Data Supplement). The checklist comprises 10 questions which address coherence and appropriateness of aims, objectives, and methodology.^20^ Quality of studies was not used as part of study selection for this review but instead to determine the current state of the evidence underpinning this area of inquiry.

### Data Analysis

Inductive framework synthesis was used, chosen as it promotes understanding of the phenomenon of interest, and ensures novel findings concerning experiences of women during breast and cervical cancer screening can emerge.^21-23^ All studies were imported to NVivo, and the findings from included studies were coded for analysis. Inductive synthesis was used to derive different stages of the breast and cervical cancer screening process described across included studies and derive an initial framework. These stages of the screening process were then used to organize findings from included studies, aligning reports and experiences of women across the different stages. The codes and stages that emerged during analysis were regularly discussed between the authors, and a final framework was agreed upon.

## RESULTS

**FIG 1:**
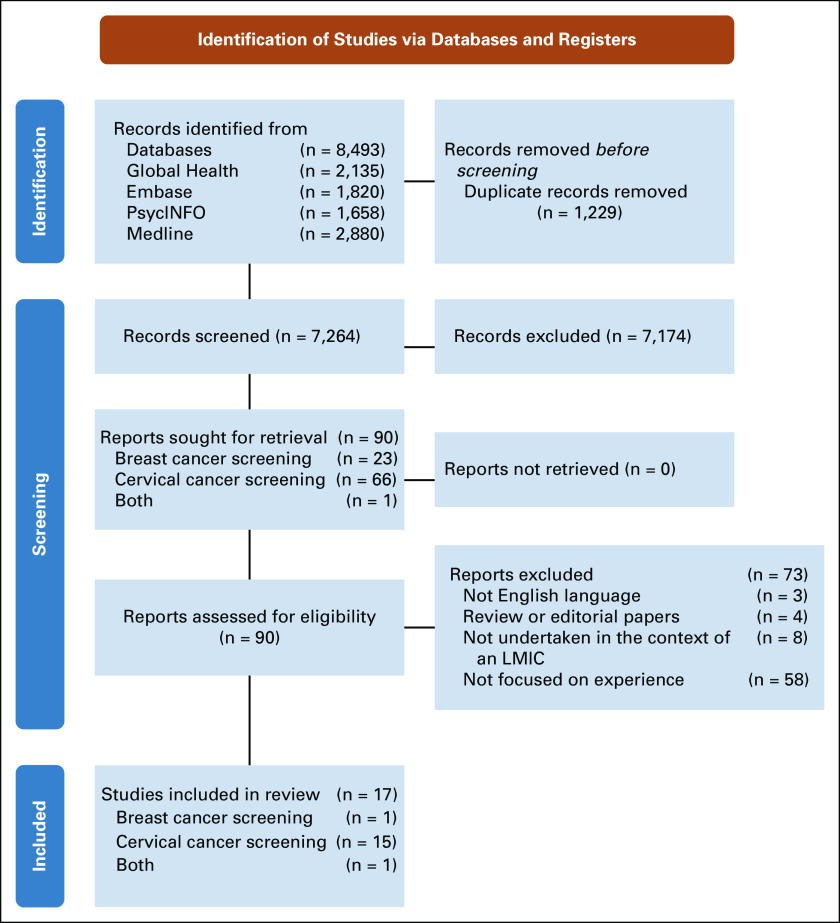
Preferred Reporting Items for Systematic Reviews and Meta-analyses flowchart. LMIC, low- and middle-income country.

The search yielded 7,264 studies, 90 of which were potentially eligible for inclusion after title and abstract screening. After full-text review, 17 studies containing a total of 722 participants were included in this review (see Fig 1 for the PRISMA flowchart). A summary of included studies is outlined in Table 1. The risk of bias across the studies was rated mostly as low across the quality criteria (see Data Supplement).

**TABLE 1:**
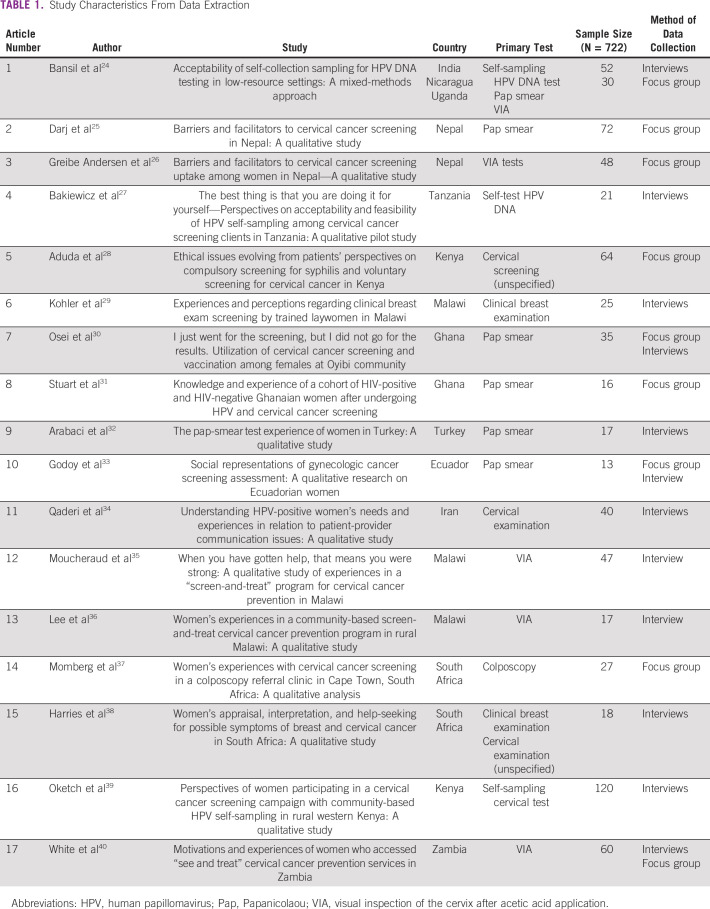
Study Characteristics From Data Extraction

Four stages were generated from the analysis of the experiences of women during breast and cervical cancer screening: (1) preparation, readiness, and engagement with screening; (2) undergoing the procedure by professional or self; (3) receipt of test results and treatment plan/outcomes; and (4) opinions on further engagement with screening.

**FIG 2:**
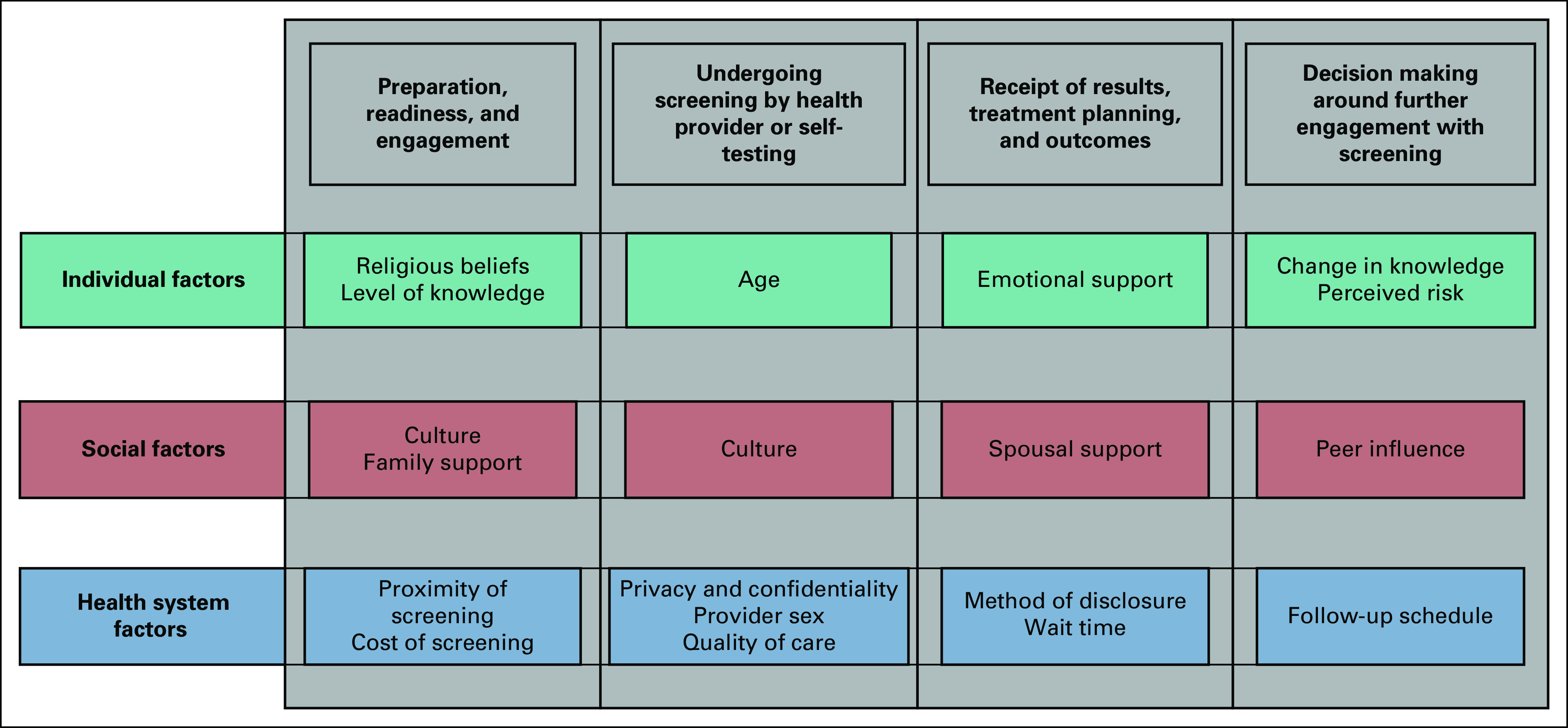
Summary of factors influencing women's experiences across four phases of breast and cervical cancer screening.

Figure 2 illustrates key elements reported by women across the four stages of screening program involvement, further stratified at the levels of individual, social, and health system factors.

### Preparation, Readiness, and Engagement With Screening

Across studies, common reasons for testing for cervical cancer included determining the presence of cancer and to access early treatment.^29,30,32^ For breast cancer screening, a participant's desire for early detection could be derived from knowing a person who has been diagnosed with breast cancer and undergone treatment, such as a mastectomy.^29^ A further reason that steered participants to engage with screening were predisposing factors such as genital warts and other health conditions (eg, infertility).^30,34^

I believe that you going to get screened and vaccinated will reduce the number of women who are infected with cervical cancer since the screening helps to detect cervical cancer. You can receive treatment when it is detected early (Ghana).^30^

At the individual level, two factors were reported that facilitated engagement. Alongside the level of knowledge on cervical or breast cancer and the importance of screening, religion and family support played a role in participants quest to seek answers concerning their health.^25,32^

Everything may happen, God knows. We do not invite illnesses, I mean they happen (Turkey).^32^

Additionally, in places where the cost of the screening services was low or no charges existed, women were willing to undergo screening.^26,30^ The proximity of screening centers encouraged others to participate in the screening.^26^

Counseling was commonly provided before screening procedures. Across studies, the content of counseling was similar; women were given information about cervical cancer, the procedure, and the options after results.^26,28,30,36,40^ Most of the women felt that they received adequate counseling on the procedure and treatment options in both breast and cervical cancer screening.^29,36,40^ Counseling was usually delivered verbally, although, in one study, women were shown videos and images and were disturbed by the description of the colposcopy procedure.^34^ There was no difference in counseling whether it was conducted by qualified health personnel (eg, nurses, doctors) or trained laywomen (ie, breast health workers).^36^

They explained to us everything that was going to happen in the screening procedure, so we knew that it's either we will be found with cancer or not. If you have been found with the cancer cells, they have equipment which they use for thermocoagulation, they explained everything beforehand (Malawi).^36^

However, negative aspects of counseling were reported. Some women felt that they were not allowed to discuss personal concerns about their sexual health because of cultural sensitivities and a lack of privacy when information was being provided to them.^25,34^

### Undergoing Screening Procedures by Health Professionals or by Self-testing

Differences arose in the reported experiences and practices for screening procedures. Women in Ghana, Kenya, and Tanzania reported that the procedure felt both private and confidential whether it was performed by themselves (eg, HPV self-sampling) or by a health professional.^26,27,30,39^ This sense of discretion made the women feel comfortable during the procedure.

The best thing [ed. about the self-sampling] is that you are doing it for yourself. […] By doing it for yourself it feels comfortable because it is you (Tanzania).^27^

Conversely, women in Nepal and Iran described multiple women being examined in the same room with no privacy and being exposed during the testing.^26,34^ Some Zambian women objected to exposing themselves to the procedure because they deemed it was not culturally acceptable.^40^

Yes, in one room; one was lying here (pointing) another was lying there (pointing). Four women were screened by four doctors. Women who went with us for the screening went home as they did not like that place (…) Three, four women were screened at the same time (Nepal).^26^

For women undergoing cervical cancer screening, the presence or absence of pain was a central and common component of the screening procedure experience. Participants in 10 of 14 studies referred to this aspect of the procedure. Women who underwent VIA or Pap smears reported experiencing intense pain or discomfort associated with the insertion of the speculum or brush.^33-36^ In Ecuador and Nepal, the discomfort was associated with embarrassment during positioning for the procedure.^25,33^

It's scary, it hurts, and it's suspicious putting yourself there with your legs open, I wish there were another way (Ecuador).^33^

Women who performed self-sampling reported little to no pain associated with the procedure.^24,27^ A minority of women in Malawi experienced mild bleeding after the self-sampling.^36^

Preference for the gender of those performing the cervical cancer screening procedures was reported across included studies. Most women mentioned a preference for female professionals because they felt shy or uncomfortable with male health providers.^25,26,28-30^ Some women completely refused to be tested or changed their presenting symptoms when they were seen by male providers.^25^ Similarly, for women undergoing breast examinations, female providers were preferred.^29^ In Ecuador and Malawi, there was not an outright preference for female professionals, with some women preferring male professionals because they were reported to be more sympathetic to their needs.^35,41^ Age-related patterns were reported too, with older women in Malawi preferring to have female providers while younger women did not have a strong preference for male providers or female providers.^35^

There were different perceptions relating to the quality of screening services between breast and cervical cancer screening. In Malawi, there were reports of women being turned away from cervical screening appointments because of a shortage of required equipment.^35^ In South Africa, participants who accessed cervical screening complained of prolonged waiting times and prejudiced health staff which negatively affected their experience, while others who had breast screening had an overall positive experience.^38^ Turkish women reported positive experiences of cervical cancer screening, reporting the use of hygienic equipment and timely and fast care received.^32^

### Receipt of Results, Treatment Planning, and Outcomes

Women reported feeling anxious while awaiting results,^30–32,42^ where reported wait times could vary from 1 to 6 months, leading to some participants feeling depressed.^37^ In Malawi, the prompt treatment led to women feeling relieved and less anxious.^36^ Anxiety relating to receiving results was compounded by how results were delivered by a health provider, although accompanying results with treatment options could promote satisfaction with care and relief from anxiety.^28,34,36^ Health professional engagement could influence anxiety, with women in Iran reporting doctors were dismissive of their reactions during the disclosure of positive test results.^34^

I was so scared. My doctor said: cancer patients don't mourn like you. HPV is not that important. I think insomuch she sees cancer it's gotten trivial for her (Iran).^34^

In Iran and Kenya, some women did not get the opportunity for their concerns about the results to be addressed.^28,34^ Those who received negative results in Kenya felt side-lined as they were not given instructions on any subsequent follow-up and engagement with screening procedures.^28^ In Ghana, women reported not being contacted to receive their results despite providing contact information to providers.^30^ For those who received results, the mode of disclosure influenced women's experiences, with verbal being preferred over written.^28^ Implications of written modes were highlighted in South Africa, with some women receiving written results and having privacy breached by their neighbors.^37^ Furthermore, those who received letters containing results did not fully understand the implications of the results.^37^ After receiving results, some women opted to receive immediate treatment while others postponed it to get spousal approval since abstinence was required after treatment.^36^

### Decision on Further Engagement With Screening

In Malawi and South Africa, women who had positive experiences during the screening program had intentions to reengage with screening procedures in the future.^35^ Women with predisposing factors for cervical cancer, such as being sexually active or having a positive HIV status, also mentioned the intention to engage with screening in the future.^35^ Women with positive experiences also mentioned the likelihood that they will inform other women in their community.^30,39^

I will recommend it to all women to get screened for it and also to get the vaccine because sex is common among the young women of today, so it will be good if they know their status (Ghana).^30^

Conversely, in Iran and Ecuador, negative experiences of pain and the attitude of providers deterred women from follow-up, treatment, or further engagement with screening.^34,43^

I no longer go to screening due to the intense pain I experienced (Iran).^34^

The dismissive and prejudiced nature of certain providers discouraged women from participating in screening in the future.^34,38^

You must sit in line—they won't explain things to you(…). I told myself I would never set foot here again (South Africa).^38^

## DISCUSSION

This study sought to explore the experiences of women during breast and cervical cancer screening and its influence on subsequent engagement.^29^ From the 17 included studies, findings highlight that screening experiences are influenced across the individual, social, service delivery, and health system levels. Initial screening experiences may influence subsequent engagement, influenced by communication and compassion of health care professionals, satisfaction with support and counseling, the levels of physical discomfort during procedures, and the duration of time and related anxiety when awaiting test results. To overcome the challenge of low uptake and coverage of screening for breast and cervical cancers in LMICs, it may be necessary to draw on experiences reported across the literature to develop screening programs that align with women's individual, social, and cultural needs.^31^

A multitude of factors was identified at the individual level. A common driver of participation in screening was the level of knowledge concerning predisposing factors, potential benefits of screening, and information gathered from people diagnosed with cancer. Previous literature has indicated that the perception of risk, severity of results, benefits, and barriers can affect adherence to screening.^44^ Although many studies have investigated the level of knowledge of women on breast and cervical cancer screening, knowledge does not necessarily translate to increased uptake.^31^ The most frequently used intervention to increase uptake in Africa is context-specific educational campaigns.^45^ In Nigeria and among Hispanic women, uptake has been shown to increase when educational campaigns were performed by peer educators or community health workers.^46-48^ The effectiveness of this strategy was attributed to the existing relationship between eligible women and the previous attendees who were peer educators.^45^ Exploring ways of using and augmenting the role of community health workers in LMICs may be a sustainable approach to facilitating engagement with screening programs.^47^

Convergence of personal and social factors occurred when considering preferences around exposure during procedures. Women's religious and sociocultural beliefs influenced feelings of embarrassment, discomfort, and preferences for female providers to perform screening procedures. A certain level of exposure may be deemed unacceptable where the practitioner is not also female,^49^ and this may affect the experience of privacy and comfort during the screening procedure. Existing studies have highlighted similar preferences for female practitioners for gynecological procedures.^50,51^ Indeed, the preference for practitioner sex in some participants can be valued higher than their skillset,^50^ although this was not reflected in studies included in this review. Culturally sensitive screening procedures should be used to address concerns about provider sex, privacy, and comfort of participants. For example, when amendments were applied to tailor a breast screening program to the cultural expectations and practices of minority Korean American women, coverage improved.^52^

The need for spousal or family support during decision making to seek care, treatment, and follow-up were evident across the included studies. Some women who decided to undergo screening were encouraged by their spouses and families, while treatment and follow-up could be hindered by spouses who did not understand their relevance. Diminished support from a spouse can reduce the likelihood of engagement with screening.^53,54^ The influence of a spouse extends to financial and emotional support, often sought from preparation through to treatment and follow-up stages.^55^ Furthermore, where a spouse has a higher level of knowledge concerning the benefits of cancer screening, their spouse is more likely to participate in screening programs.^56^

The interaction between providers and participants as part of service delivery was reported as valued by participants. Women reported various emotions during screening, and procedures were known to evoke anxiety, worry, and fear of the possible outcomes.^57^ One effective way to address these emotions and misconceptions is through pretest and post-test counseling.^58^ Experiences associated with pain, discomfort, or dissatisfaction with services can lead to fear-mongering and thereby discourage prospective participation in screening.^59^

With regards to health systems, logistical factors such as overburdened or distant facilities and lack of equipment affected the quality of care provided to participants. Accessibility of screening facilities influenced the decision to undergo screening.^60,61^ Approaches to increasing the accessibility of programs include mobile screening services in hard-to-reach areas, such as mobile cervical and breast cancer screening services in Brazil.^62^ In Botswana, a telemedicine approach was adopted where community workers used mobile phones to send images of the cervix after VIA as part of a consultation with gynecologists in areas where gynecologists were not available.^63^ Similarly, telemedicine has been successfully used in rural India for screening oral and cervical cancers.^64^ A combination of mobile services where services are brought to eligible people and telemedicine may help improve the quality of care and reduce accessibility barriers. Furthermore, the integration of reproductive health services has been proposed as a cost-efficient way of increasing coverage of some screening services.^65^ Integration is favored because it promotes comprehensive management of high-risk individuals and uses pre-existing channels (Joint United Nations Programme on HIV/AIDS, 2016). Peer educators and community workers have also successfully supported the delivery of family planning and HIV services.^46^ In South Africa, a pilot combining HIV and reproductive health services for female sex workers was successful in increasing screening program utilization.^66^ Many pilots of integration have been conducted yet few have been scaled up to the national level.^66^ In a small number of countries, including Kenya and Uganda, cervical cancer screening has successfully been integrated into either family planning or HIV services.^65^

Table 2 draws together the key findings from the systematic review, evidence of interventions that influence uptake and engagement with screening procedures, and related recommendations.

**TABLE 2:**
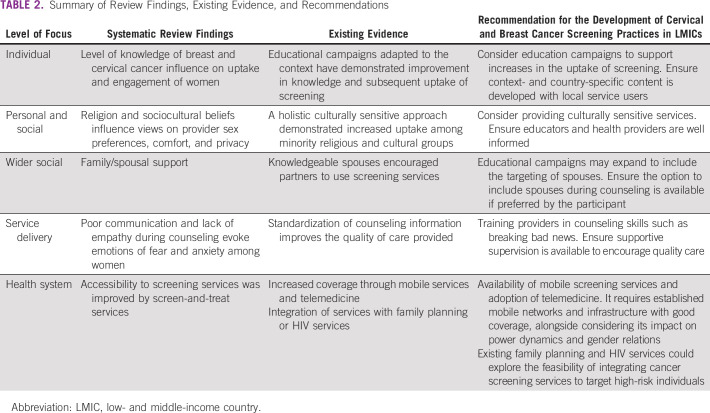
Summary of Review Findings, Existing Evidence, and Recommendations

A broad search strategy was used; however, the existing literature identified included a lack of diversity across LMICs, with a notable prominence of studies from SSA over other regions. There was also an imbalance in the representation of women reporting experiences of breast cancer screening with only one included study. This highlights a need to increase the evidence base to increase awareness of the experiences of women undergoing breast cancer screening in LMICs. The review also limited studies to those in the English language, so the review may not fully report the breadth of findings available across the research literature. Authors of primary studies were not contacted for further information because of time limitations; therefore, relevant information may have been lost.

In conclusion, this review highlights multiple factors across the individual, social, and health systems levels that can influence women's experiences of breast and cervical cancer screening programs in LMICs. Our findings were used to generate evidence-informed recommendations to guide refinements to the delivery of screening programs for women with breast and cervical cancers. Ensuring positive and continued engagement with screening programs may be an essential component in supporting reductions in the disease and mortality burden of breast and cervical cancers in LMICs. Further research will be required to determine optimal approaches to the implementation of the recommendations and their effectiveness in improving the experience of screening.

## Data Availability

Data sets generated as part of this systematic review, including the eligibility coding of title and abstracts and full-text review of articles, are available on request to the corresponding author.
